# Telemonitoring and Health Counseling for Self-Management Support of Patients With Type 2 Diabetes: A Randomized Controlled Trial

**DOI:** 10.2196/diabetes.6884

**Published:** 2017-06-26

**Authors:** Inger Lindberg, Astrid Torbjørnsen, Siv Söderberg, Lis Ribu

**Affiliations:** 1 Division of Nursing Department of Health Science Luleå University of Technology Luleå Sweden; 2 Faculty of Medicine Department of General Practice, Institute of Health and Society University of Oslo Oslo Norway; 3 Faculty of Health Sciences Department of Nursing and Health Promotion Oslo and Akershus University College of Applied Sciences Oslo Norway; 4 Department of Nursing Sciences Mid Sweden University Östersund Sweden

**Keywords:** self-management, clinical trial, telemonitoring, type 2 diabetes, health-related quality of life

## Abstract

**Background:**

The prevalence of diabetes is increasing among adults globally, and there is a need for new models of health care delivery. Research has shown that self-management approaches encourage persons with chronic conditions to take a primary role in managing their daily care.

**Objective:**

The objective of this study was to investigate whether the introduction of a health technology-supported self-management program involving telemonitoring and health counseling had beneficial effects on glycated hemoglobin (HbA_1c_), other clinical variables (height, weight, body mass index, blood pressure, blood lipid profile), and health-related quality of life (HRQoL), as measured using the Short Form Health Survey (SF-36) version 2 in patients with type 2 diabetes.

**Methods:**

This was a pragmatic randomized controlled trial of patients with type 2 diabetes. Both the control and intervention groups received usual care. The intervention group also participated in additional health promotion activities with the use of the Prescribed Healthcare Web application for self-monitoring of blood glucose and blood pressure. About every second month or when needed, the general practitioner or the diabetes nurse reviewed the results and the health care activity plan.

**Results:**

A total of 166 patients with type 2 diabetes were randomly assigned to the intervention (n=87) or control (n=79) groups. From the baseline to follow-up, 36 patients in the intervention group and 5 patients in the control group were lost to follow-up, and 2 patients died. Additionally, HbA_1c_ was not available at baseline in one patient in the intervention group. A total of 122 patients were included in the final analysis after 19 months. There were no significant differences between the groups in the primary outcome HbA_1c_ level (*P*=.33), and in the secondary outcome HRQoL as measured using SF-36. A total of 80% (67/87) of the patients in the intervention group at the baseline, and 98% (47/50) of the responders after 19-month intervention were familiar with using a personal computer (*P*=.001). After 19 months, nonresponders (ie, data from baseline) reported significantly poorer mental health in social functioning and role emotional subscales on the SF-36 (*P*=.03, and *P*=.01, respectively).

**Conclusions:**

The primary outcome HbA_1c_ level and the secondary outcome HRQoL did not differ between groups after the 19-month follow-up. Those lost to follow-up reported significantly poorer mental health than did the responders in the intervention group.

**Trial Registration:**

Clinicaltrials.gov NCT01478672; https://clinicaltrials.gov/ct2/show/NCT01478672 (Archived by WebCite at http://www.webcitation.org/6r4eILeyu)

## Introduction

Research shows that self-management approaches encourage persons with a chronic condition globally to take a primary role in managing the daily care of their illness [1]. Through self-management interventions, patients with type 2 diabetes are equipped with essential skills to participate actively in self-management behavior and to manage their condition successfully [2]. The world prevalence of diabetes among adults (aged 20-79) was estimated at 415 million in 2015 (8.8%) and is expected to reach 642 million by 2040 (10.4%). Persons with type 2 diabetes constitute 95% of all cases. It is estimated that more than 59.8 million persons in the European region have diabetes, and that this number will rise to 71.1 million by 2040 [3]. In Sweden alone, there were 446,900 cases of diabetes in adults in 2015 (6.3%), with an estimated 168,700 undiagnosed cases [4].

In light of the increasing incidence of diabetes, preventive measures and lifestyle modifications are undeniably of utmost importance. Keeping blood glucose levels under control can reduce the complications of diabetes [5,6]. The prevention of complications is important and includes lifestyle management such as changing diet and participating in regular physical activity [7-9]. Studies show that persons with diabetes fear long-term complications that may influence their quality of life [10,11].

Self-management is recognized as a key component in the clinical treatment of diabetes, but patients often lack the knowledge and skills needed to manage their condition on a daily basis. The inability to understand the fundamental influences of diabetes-management activities on overall glycemic control leads to low levels of participation in self-care behaviors [12].

Systematic reviews [13-16]have provided evidence that telehealth interventions have a positive effect on the control of blood glucose levels in persons with diabetes. Home telehealth interventions reduce the number of patients hospitalized and the number of bed days of care, and are similar or favorable to the usual care in terms of quality of life, patient satisfaction, and adherence to treatment for persons with diabetes and chronic conditions [15]. Studies [14-18] indicate that home telehealth interventions are similar or favorable to the usual care in terms of quality of life, patient satisfaction, and adherence to treatment for people with diabetes and chronic conditions. Furthermore, Ciemins et al [19] reported that telehealth is an effective mode for providing diabetes care to rural patients when compared with face-to-face visits.

Despite these positive research results, home telemonitoring has also produced contradictory results, and the addition of technology alone does not improve the outcome of glycated hemoglobin (HbA_1c_) for persons with type 2 diabetes [20-22]. One study has argued that health practices need to be selective in the use of telemonitoring, by limiting it to patients who have the motivation or a significant change in care such as starting insulin [20]. Earlier research also indicates that many patients who voluntarily participate in a telemedicine study are actually in a pre-action stage for behavioral change in the start-up phase, but they may not be ready to make changes in diet and physical activity [23].

A high dropout rate in telemedicine studies is not unusual; thus, it is important to report the discontinuation rate and/or being lost to follow-up in these studies [24]. Therefore, practices need to understand both the capabilities and limitations of the technology, as well as the involvement of the patients and stakeholders, and their willingness to use the tools. Telemedicine interventions in diabetes care have earlier evaluated the use of different telemedicine tools, the interaction between the technology and users [14], and the use of telemedicine with or without support from health care provider [25-29]; however, more research is needed.

Evaluating the clinical effectiveness of telemedicine is another important area [30]. Health-related quality of life (HRQoL) is a crucial outcome for persons living with a chronic condition, because it measures the impact of the condition on daily life. Therefore, evaluating the success of self-management interventions in terms of improvements in HRQoL seems appropriate from the perspective of persons with a chronic condition [31]. Quality of life for persons with diabetes is thought to be affected primarily by vascular complications such as peripheral vascular disease, cardiovascular disease, or associated comorbidities [32]. However, research indicates that functional impairments and physical disability affect the HRQoL of older persons with diabetes most significantly [32-34]. Therefore, the focus of diabetes management should be on the overall well-being rather than on the biological control of diabetes alone [35].

Thus, the objective of this study was to investigate whether the introduction of a Web application “Prescribed Healthcare” for self-monitoring of parameters such as blood glucose level and blood pressure, together with health counseling, produced benefits in terms of HbA_1c_ level; other clinical variables such as height, weight, body mass index (BMI), blood pressure, and blood lipid profile; and HRQoL in patients with type 2 diabetes.

## Methods

### Design and Setting

This study was a pragmatic parallel-group, unblinded, randomized controlled trial [36] with 1 intervention group and 1 control group. The study had a longitudinal design with 2 assessment points: at baseline and at the end of the trial (after 19 months). This is a Swedish study as part of the European Union collaborative project called Renewing Health (RH). The overall aim of the RH project was to evaluate innovative telemedicine tools [30]. The Swedish part of the project was conducted in 4 health care centers situated in the northern part of Sweden during the years 2011-2013. This northern part covers 25% of Sweden’s land area and has a population of 250,000 inhabitants.

### Sample

A sample of 166 patients with type 2 diabetes was included in the study. The inclusion criteria were: having type 2 diabetes diagnosed >3 months before enrollment, HbA_1c_ level >6.5%, age ≥18 years, the capability to complete the questionnaires and to use the devices provided, and being cognitively able to participate. The patients who met the inclusion criteria were recruited from their health care center and approached through an information letter sent from their health care center in May 2011.

For sample size calculations, we needed 63 individuals in each group to maintain a statistical power of 80% and a significance level of 5%, and a standard deviation (SD) of the outcome variable of 0.5. Assuming a dropout rate of 20%, our aim was to enroll 95 individuals in each group to ensure that we had sufficient statistical power to reveal a significant difference of ≥.1 or in the primary outcome HbA_1c_ level to be statistically significant.

### Usual Care

The control group received usual care. Care for patients with type 2 diabetes was regulated by the Swedish national guidelines for the treatment of diabetes mellitus and included methods for implementing lifestyle change, medical treatment, and follow-up [37]. Foot inspection was also recommended. In accordance with the guidelines, an ophthalmologist was responsible for eye examinations. All patients with type 2 diabetes were given a glucose meter, test strips, and lancets at no charge from the County Council. A multidisciplinary health counseling team comprising a general practitioner (GP), physiotherapist, dietitian, and diabetes nurse support the patients in performing physical activities and adopting a healthy diet. Patients with type 2 diabetes self-monitor their blood glucose level and report the results to their diabetes nurse.

### The Intervention

**Figure 1:**
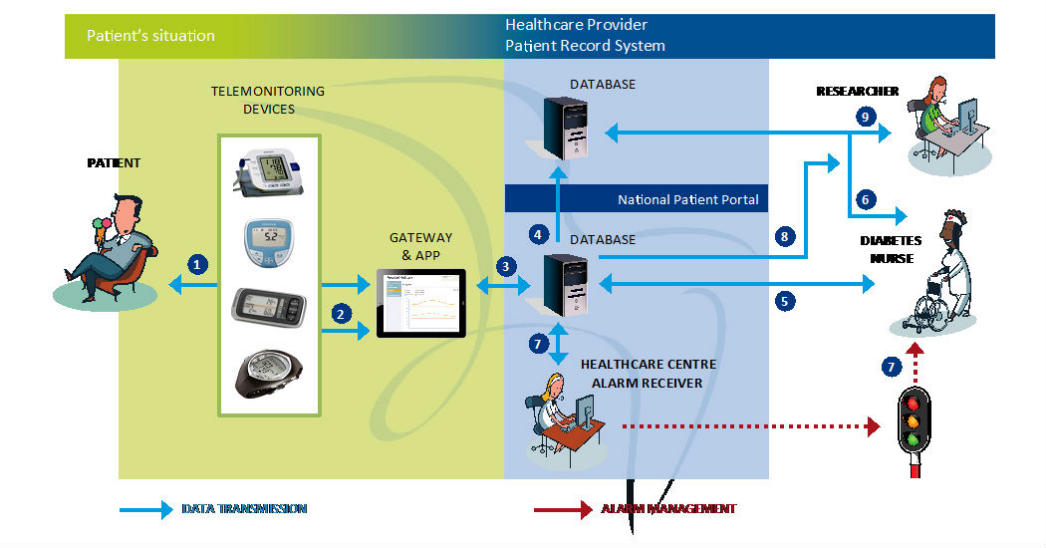
Telemonitoring devices and information flow during the field trial.

The intervention group received usual care in addition to the intervention, using a method that combined health counseling with the National Patient Portal. The idea was that the patients became more actively involved by self-monitoring their health by this Web-based management application. Measurement equipment, such as a blood glucose meter, was provided to the patients, and they used their own personal computers (PCs) to communicate. The few patients with no access to a computer were provided a tablet computer (see Figure 1).

Figure 1 shows how the patient is authenticated through electronic identification (1) to obtain access to the National Patient Portal. The connection between the patient and the National Patient Portal is encrypted (2). When the patient selects the Web application “Prescribed Healthcare,” a connection is established to the servers at the County Council of Norrbotten. This connection (3) is made through a secure computer network connecting all health care providers in Sweden (Sjunet), and the connection is established with the Prescribed Healthcare server through configured firewalls (4). The caregivers are authenticated through SITHS, which is a smartcard-based secure authentication for caregivers employed by Swedish health care providers (6), and routed to the caregivers’ intranet for the County Council of Norrbotten (7). The caregivers can obtain access to the electronic medical record system (8), connect to the National Patient Portal (3), and be rerouted to the Prescribed Healthcare server (4). Each health care center has an alarm receiver. If the health care provider who prescribed the measurement equipment does not manage the alarm on time, it is sent to another health care provider who can manage the alarm on time.

A patient entering the intervention, started with group sessions at the health care center that aimed to educate and motivate the patient to perform lifestyle modifications such as increasing physical activity, adopting a healthy diet, stopping smoking, and reducing alcohol consumption. The patient was trained to use the technology, to manage his or her health information, and to interact with health care professionals via email or video through the Prescribed Healthcare. An individualized activity plan was developed for each patient in the intervention group. During the project period, the patients performed health promotion activities and self-registered their parameters, such as duration of physical activity, into the application. The health care professionals provided reference values, and when applicable, the alarm levels.

The patients measured and manually entered medical parameters such as blood glucose level and blood pressure, which could be viewed through intuitive diagrams in each patient registration. The reference values made it easier for patients and caregivers to evaluate the outcome. If an alarm level was reached, the health care professional was notified. About every second month, or when needed according to the patient’s initiative, the GP and/or diabetes nurse reviewed the results and revised the health care activity plan as needed. The patients then received feedback, such as any changes in medication or supporting comments about the performance of physical activities, from the health care professional via email or video. Furthermore, GPs, diabetes nurses (Figure 1), physiotherapists, and nutritionists worked cooperatively to interact with and manage each patient.

### Randomization

In May 2011, eligible patients who met the inclusion criteria were approached with an information letter sent from their health care center. After the patient signed an informed consent form and completed the questionnaires, single-blind randomization was performed following standard procedures with a PC-based generation of random sequences, and an allocation based on consecutive assignment. A statistician performed the randomization; he or she had no access to the participants’ personal code numbers. The researchers handling the database had no access to the participants’ personal code numbers, when they analyzed the data.

### Sociodemographic Measures

Demographic characteristics were collected from the health care centers before randomization through self-reported questionnaires. These included date of birth, gender, education, marital status, smoking habits, and level of computer and mobile phone skills.

### Clinical Measures

Clinical baseline measures were collected at the health care centers before randomization. The participants’ HbA_1c_ level, blood lipid profile, height, weight, and blood pressure were measured.

### Primary Outcome

The primary outcome HbA_1c_ level was measured in the control and intervention groups at the baseline and after the intervention at 19 months. The rationale for the 19-month time frame was the desire for a long follow-up intervention, and for practical reasons, to allow the data collection to finish at the same time point as the other RH trials.

### Secondary Outcome

The secondary outcome HRQoL was measured using the Short Form Health Survey (SF-36) version 2, which comprises 36 questions that measure 8 conceptual domains within physical functioning and mental health [38]. In addition to the 8 subscales, SF-36 is also analyzed as a 2-factor model, with physical and mental component summary scales. In this study, both the subscale scores and the summary scores of the SF-36 were presented. The SF-36 is internationally recognized as a reliable and valid tool [39].

### Statistical Methods

Baseline characteristics were recorded for all randomized patients and between responders and nonresponders (ie, those missing) at 19 months. All analyses were based on the intention-to-treat principle. Categorical data were reported as counts and percentages. Associations between pairs of categorical variables were analyzed using the chi square test; continuous data were described as mean and SD (when normally distributed), or as median, minimum, and maximum (when not normally distributed). Group differences were identified using the Mann-Whitney *U* test. An independent sample *t* test was used to compare the change in the primary outcome between the intervention and control groups from the baseline to the follow-up at 19 months. All tests were two-sided. *P* values of ≥.05 were considered to be significant. SPSS Statistics version 22 (IBM Corp) was used for all analyses.

### Ethics and Safety

The study was conducted according to the Ethical Review Act [40] and was approved by the Regional Ethical Review Board, Umeå, Sweden (Dnr 2010/386-31M). The portal provides secure access to their health information for all Swedish citizens and supports electronic interactions with health care professionals.

## Results

**Figure 2:**
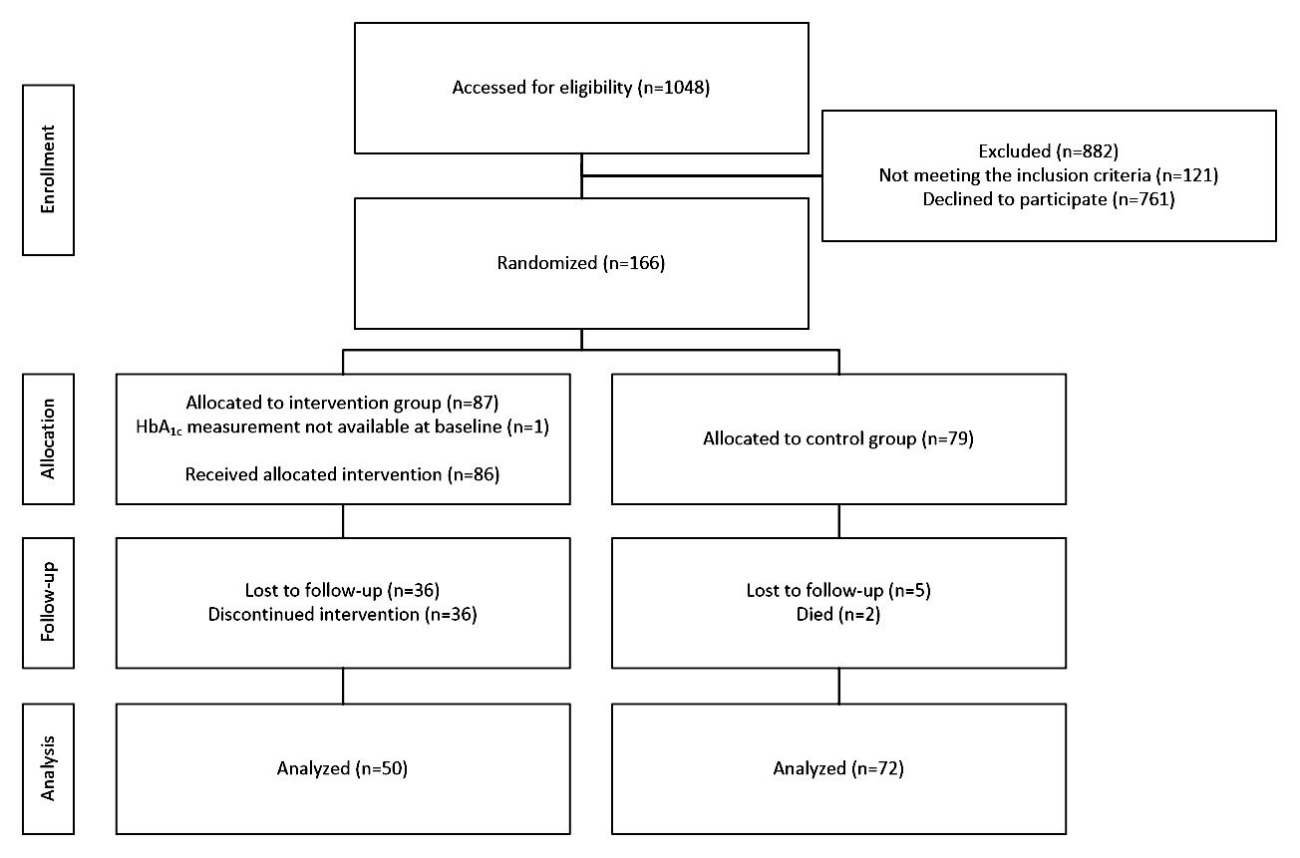
Flow diagram depicting the phases of the parallel randomized trial of the two groups (intervention and control group).

Of 1048 eligible patients, 121 (11.55%) did not meet the inclusion criteria, and 761 (72.61%) chose not to participate in the study. A total of 166 patients (15.84%) were included for randomization; 87 patients were randomly assigned to the intervention group and 79 patients to the control group (Figure 2). One patient randomized into the intervention group was removed from the analysis because of a missing HbA_1c_ value. The percentages of dropouts differed between the groups; the reasons for dropping out included: being too ill, changing health care centers, or feeling that the technology was too difficult to handle. From the baseline to the follow-up, 36/86 patients (42%) in the intervention group and 5/79 patients (6%) in the control group were lost to follow-up; 2/79 patients (9%) died (Figure 2).

Of the 166 patients included in this study, 122 were included in the final analysis after 19 months. Their mean age was 67.5 years (SD 9.3), 48 (29.1 %) of the patients were female, and 63 (38.2 %) had >12 years of education. The mean HbA_1c_ was 64.6 mmol/mol (SD 11.0)/8.1% (SD 1.0), and the mean BMI was 30.7 kg/m^2^ (SD 5.0). Twenty patients (12.1%) reported 2 or more comorbidities. The baseline data are presented for all patients in Table 1. This table also presents the data for the responders in both groups at 19 months. The baseline clinical and demographic characteristics did not differ significantly between the intervention and control groups (Table 1). Because of the high attrition rate and the large number of nonresponders in the intervention group (Figure 2), we investigated possible differences between responders and nonresponders in the intervention group, by comparing the values after 19 months with the baseline data (Table 1).

### Primary Outcome

Only patients, who responded at 19 months, and for whom the HbA_1c_ level was measured and available, were included in the analysis of the primary and secondary outcomes. The numbers analyzed were 50 patients in the intervention group and 72 patients in the control group (Figure 1).

**Table 1:** Participants’ characteristics at the baseline, and at 19 months for HbA_1c_ responders.

Characteristics		All randomized at baseline		Responders at the 19-month follow-up	
		Intervention (n=86)	Control (n=79)	Intervention (n=50)	Control (n=72)
**Age in years**					
	Mean (SD)^a^	66.8 (8.8)	68.3 (9.9)	64.8 (8.5)	68.8 (9.8)
	Median	67	69	66	70
	Range	(39-91)	(37-89)	(39-91)	(37-89)
	Missing	2	0	2	0
**Gender: female**					
	n (%)	24 (29)	24 (30)	10 (21)	21 (29)
	Missing	2	0	2	0
**Education: >12 years**					
	n (%)	31 (37)	33 (43)	21 (45)	28 (41)
	Missing	3	4	3	3
**Marital status: married**					
	n (%)	47 (56)	49 (61)	30 (63)	45 (63)
	Missing	2	1	2	0
**Height in cm**					
	Mean (SD)	170.5 (9.6)	171.3 (8.2)	171.7 (8.2)	171.4 (8.1)
	Median	171	171.5	172	171.8
	Range	(151.5-190.5)	(153-192)	(156-189)	(153-192)
	Missing	1	0	0	0
**Weight in kg**					
	Mean (SD)	90.3 (16.3)	89.3 (16.9)	93.0 (17.8)	88.7 (14.6)
	Median	88.0	90.8	92.1	91
	Range	(55.0-124.8)	(58.7-156.0)	(55-124.8)	(58.7-131.4)
	Missing	1	0	0	0
**BMI^b^ in kg/m^2^**					
	Mean (SD)	31.0 (4.8)	30.4 (5.2)	31.4 (4.9)	30.2 (4.4)
	Median	30.3	30.0	31.3	30.1
	Range	(17.8,-40.7)	(20.8,54.0)	(17.8,40.7)	(20.8,43.3)
	Missing	1	0	0	0
**HbA_1c_^c^ in mmol/mol**					
	Mean (SD)	65.3 (11.7)	63.9 (10.2)		
	Mean (SD)	65.3 (11.7)	63.9 (10.2)	65.8 (11.8)	63.6 (10.0)
	Median	62.5	61.0	63	61.5
	Range	(53.0-108.0)	(53.0-96.0)	(53-108)	(53-96)
	Missing	0	0	0	0
**HbA_1c_ in %**					
	Mean (SD)	8.1 (1.1)	8.0 (0.9)	8.2 (1.1)	8.0 (0.9)
	Median	7.9	7.7	7.9	7.8
	Range	(7.0-12.0)	(7.0-10.9)	(7.0,12.0)	(7.0,10.9)
	Missing	0	0	0	0
**Comorbidities: 2 or more**					
	n (%)	8 (9)	12 (15)	3 (6)	10 (14)
	Missing	0	0	0	0
**Smoking: yes**					
	n (%)	14 (17)	12 (16)	9 (18)	11 (16)
	Missing	3	2	2	1
**Use of PC^d^: yes**					
	n (%)	67 (80)^e^	57 (72)	47 (98)^e^	52 (72)
	Missing	2	0	2	0
**Use of cell phone: yes**					
	n (%)	79 (94)	72 (92)	47 (98)	66 (92)
	Missing	2	1	2	0

^a^SD: standard deviation.

^b^BMI: body mass index.

^c^HBA_1c_: glycated hemoglobin.

^d^PC: personal computer.

^e^The difference between randomized participants and responders due to use of PC are statistically significant with *P*=.001 (chi-square test).

**Table 2:** HbA_1c_ responders: clinical characteristics.

Measures		All randomized at baseline		Responders at the 19-month follow-up	
		Intervention mean (SD)^a^	Control mean (SD)	Intervention mean (SD)	Control mean (SD)
**HbA_1c_^b^ in mmol/mol**		65.8 (11.8)	63.6 (10.0)	64.5 (15.8)	64.6 (14.8)
	Missing	0	0	0	0
**HbA_1c_ in %**		8.2 (11)	8.0 (0.9)	8.0 (1.4)	8.1 (1.4)
	Missing	0	0	0	0
**BMI^c^ in kg/m^2^**		31.4 (4.9)	30.2 (4.4)	31.1 (4.6)	29.8 (4.6)
	Missing	0	0	0	1
**Blood pressure in mm Hg**					
	Diastolic	146 (19)	145 (17)	143 (24)	144 (18)
	Systolic	83 (10)	81 (11)	85 (12)	82 (8)
	Missing	0	0	0	0
**Lipids**					
	S-cholesterol^d^	5.29 (1.04)	5.35 (1.38)	5.18 (1.11)	5.21 (1.14)
	Missing	0	0	0	0
	Triglycerides	2.09 (1.23)	2.35 (1.75)	1.99 (0.98)	2.13 (1.25)
	Missing	0	0	0	0
	HDL^e^	1.23 (0.27)	1.26 (0.32)	1.22 (0.24)	1.29 (0.31)
	Missing	0	0	0	0
	LDL^f^	3.15 (0.86)	3.05 (1.19)	3.04 (0.84)	3.02 (1.01)
	Missing	4	8	3	7

^a^SD: standard deviation.

^b^HBA_1c_: glycated hemoglobin.

^c^BMI: body mass index.

^d^S-cholesterol: serum cholesterol.

^e^HDL: high-density lipoprotein.

^f^LDL: low-density lipoprotein.

We found no significant differences between the intervention and control groups in the change in HbA_1c_ level between the baseline and the 19-month follow-up (*P*=.33, 95% CI [−0.65 to 0.22]; Tables 2 and 3).

### Secondary Outcome

The changes in the domains of SF-36 from the baseline to the 19-month follow-up did not differ significantly between the intervention and control groups. Similarly, there were no significant differences in clinical variables such as blood pressure, lipid levels, and BMI (data not shown). However, within the control group BMI decreased significantly during the study (Table 3), and the domains of bodily pain and role emotional functioning measured by the SF-36 also decreased (data not shown). In addition, the domain of physical functioning as measured by the SF-36 decreased significantly in the intervention group (data not shown). These findings might be accidental and with no obvious explications.

### Other Clinical Outcomes

At the baseline, 80% (67/86) of the patients randomized to the intervention group were familiar with using a PC; after the 19-month intervention, this percentage was 98% (47/50) among the patients in the intervention group (Table 1). This difference from the baseline to the follow-up was significant (*P*=.001, chi square test; Table 1).

**Table 3:** HbA_1c_ responders: Change from baseline to 19 months.

Measures		Intervention		Control	
		Mean	95% CI	Mean	95% CI
**HbA**_1c_^a^ **in mmol/mol**		−1.36	−4.81 to 2.09	0.97	−2.20 to 4.15
	Missing	0		0	
**HbA**_1c_ **in %**		−0.12	−0.44 to 0.19	0.09	−0.20 to 0.38
	Missing	0		0	
**BMI^b^** **in kg/m^2^**		−0.35	−0.72 to 0.03	−0.36	−0.60 to −0.11
	Missing	0		1	
**Blood pressure in mm Hg**					
	Diastolic	−2.78	−8.30 to 2.74	−1.13	−4.83 to 2.58
	Systolic	2.70	−0.96 to 6.36	0.83	−1.73 to 3.39
	Missing	0		0	
**Lipids**					
	S-cholesterol^c^	−0.11	−0.34 to 0.12	−0.13	−0.40 to 0.13
	Missing	0		0	
	Triglycerides	−0.11	−0.35 to 0.13	−0.22	−0.50 to 0.05
	Missing	0		0	
	HDL^d^	−0.02	−0.08 to 0.04	0.03	−0.01 to 0.07
	Missing	0		0	
	LDL^e^	−0.11	−0.31 to 0.10	−0.09	−0.33 to 0.15
	Missing	4		9	

^a^HBA_1c_: glycated hemoglobin.

^b^BMI: body mass index.

^c^S-cholesterol: serum cholesterol.

^d^HDL: high-density lipoprotein.

^e^LDL: low-density lipoprotein.

**Figure 3:**
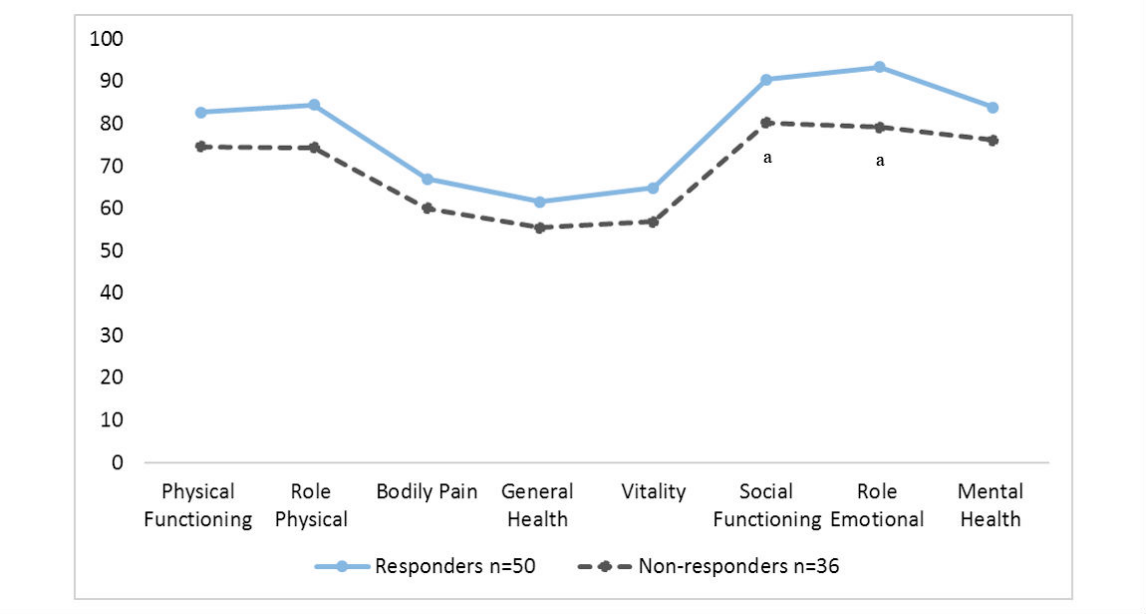
Intervention group: differences in HRQoL on the SF-36 between responders and nonresponders at 19 months (with baseline data). Significant differences were found between responders and nonresponders in social functioning and role emotional functioning (both P< .03).

We also found significant differences in some SF-36 scores between the responders and the nonresponders in the intervention group after the 19 months (with baseline data). The responders reporting higher mental health in social functioning and in role emotional functioning than nonresponders (*P*=.03 and *P*=.01, respectively; Figure 3). There was also a trend for responders to report a higher HRQoL in all SF-36 domains as compared with the nonresponders. The nonresponders had significantly higher serum cholesterol levels than the responders (*P*=.04). However, given the limited sample size, this finding might be accidental.

### Harm

No adverse events were reported during the study. Two deaths were reported in the study, both in the control group, but they were not related to the study or a lack of the intervention.

## Discussion

### Principal Findings

The results of this study show no significant changes in the primary outcome of HbA_1c_ level from the baseline to the 19-month follow-up for the patients included in the study. We found no differences in the changes in the secondary outcomes between the intervention and control groups. This is inconsistent with studies conducted in parallel with ours in the RH project [27-29,41]. One possible reason for these similarities is that the inclusion criteria and the primary outcome, Hb_A1c_ level was low (>6.5%); therefore there was little chance for improvement, which was also noted by other studies in the RH project [27-29]. However, patients whose HbA_1c_ is around 6.5% are often in need of lifestyle interventions in addition to taking medication, and such lifestyle and behavioral changes may reduce the need for or dose of medication.

When using HRQoL measures as variables, as in this study, one can expect smaller changes over time and a larger variation between the persons, which means that more patients must be included. A larger sample is often desired, but it is often difficult to recruit a sufficient sample [42]. In our study, the inclusion of participants was time-consuming. Whether the use of the SF-36 was suitable for the aim of this study can be questioned. Using disease-specific instruments to assess the special conditions and concerns of diagnostic groups could be more sensitive for detecting small changes [10] than using generic measures such as the SF-36. We discussed previously whether these primary and secondary outcome measures were suitable for measuring behavior changes and the degree of self-management, and whether primary outcomes other than HbA_1c_ could have been better suited [29].

Of importance is that this randomized controlled trial showed no significant differences between patients in the intervention and control groups at baseline, which shows that the groups were balanced at the onset. The duration of the follow-up in this study might have increased the power, although the sample size was small.

Of interest was the trend for responders to report a higher HRQoL in all SF-36 domains compared with the nonresponders (Figure 2). Nonresponders reported lower HRQoL, and had significantly lower scores on social functioning, role emotional functioning, and all HRQoL domains. HRQoL was used to evaluate the effect of a telemedicine intervention with a self-management tool and health counseling, but HRQoL can also be used to identify patients who may be in need of more support. These findings identify a nonresponder population, with more health problems, who may need greater support and follow-up than do responders, and, as such, may need greater care and may impose a larger burden on the health care system. The patients in this study were older, but with few comorbidities, although many had a high BMI and incidence of obesity. The findings indicate that nonresponders had poorer mental health at inclusion than the responders of the intervention. Earlier research has noted that psychosocial problems are common among persons with diabetes, and that these problems represent barriers for self-management [11]. eHealth is a tool that could help to reduce costs and provide a more efficient delivery of care [30]; however, this group of patients was less familiar with computers and did not respond to the intervention provided.

As previously mentioned, high dropout rates is a typical problem in self-help applications. Others have commented on the importance of addressing this phenomenon because it poses a challenge to the evaluation of eHealth applications [24]. More research designed to investigate this problem is warranted. The development phase of a complex intervention such as ours is considered to be important [43], and involves investigating both the existing evidence and the targeted group. For example, further knowledge about the targeted group may be obtained by conducting qualitative in-depth interviews in the preplanning stage of a complex intervention; this would help the investigators to learn more about user experiences and to identify both positive and negative user interactions [14]. The incorporation of user involvement (ie, patients, health care personnel, and stakeholders) is recommended at all levels in the design of telemedicine studies, as in this study [30]. This is also important to facilitate limplementation in health care organizations[43].

Of interest, we found significant differences between responders and nonresponders in the intervention group; the latter were less familiar with the use of PCs (*P*<.001; Table 2). This finding may indicate that those not accustomed to using a PC withdrew their participation in the study, and that the intervention was less likely to be accepted by patients with little experience in the use of a PC; however, if correct, the reason for this is not clear. It has been speculated that the lack of data and knowledge about withdrawal and/or dropout rates reflects a lack of investigation of this phenomenon, or that the reasons may be known but have not been published or were beyond the scope of reported trials [14]. Findings from the Whole System Demonstrator telehealth program in the United Kingdom have indicated that active rejection and patients’ lack of acceptance of the telemedicine intervention are the most frequent reasons for withdrawal. The presence of diabetes was a factor leading to greater rejection of an intervention than were other chronic diseases [44]. This could reflect that many persons with diabetes are well trained in the recording of their clinical data and that the introduction of a new system for self-monitoring is perceived as a disruption to a well-practiced regime and is therefore not acceptable. However, the reasons for withdrawing from a trial are multifaceted [44].

Research has also investigated whether there is a literacy divide between responders and nonresponders of telemedicine interventions. The effect of health literacy has been considered by earlier studies using different telehealth applications [45,46]. Health literacy can be referred to as “the cognitive and social skills which determine the motivation and ability of individuals to gain access to, understand, and use information in ways which promote and maintain good health” [47]. Our findings confirm the importance of recognizing that there is no “one size fits all” approach, meaning that when developing and initiating interventions such as ours, health care staff needs to consider carefully the patients’ health literacy; for example, being old should not be a criterion for exclusion [48]. Earlier research has emphasized the importance of developing digital interventions that are designed to be accessible, and engaging persons with a wide range of health literacy levels [49]. Improvements in health literacy outcomes after a digital health intervention depend more on a clear design and person-based intervention to establish an in-depth understanding of the views and perspectives of the targeted group, rather than on interactivity and audiovisual presentation [50]. The interactive and audiovisual elements of the intervention are especially important for motivating the participants. It has also been shown that participants without adequate education or with a low health literacy level have a lower compliance, and that active participation with support from a health care service provider can reinforce a recommended behavior [51].

The use of telehealth and eHealth applications on the Web, as in this study, is therefore not appropriate for all persons with a chronic condition. This should be taken into consideration when developing a complex intervention such as ours. Self-management interventions with innovative treatments, such as the use of health technology devices at home and in close cooperation with community health centers, could be a more suited intervention for patients who self-manage better than some of those included in this study. Therefore, it is necessary to assess the targeted group and their characteristics before developing an intervention, because they may differ significantly in needs and health status, or be in need of more intensive interventions with more advanced and tailored support from health care providers than what was offered in this study. Earlier research also indicated a need for tighter self-management support of less motivated groups among patients with type 2 diabetes participating in telemedicine research [20], and of those not yet ready to change their behavior [23].

More research is needed to identify those not responding to telemedicine intervention to understand how to design different telemedicine applications that are suitable for specific groups and to identify the kinds of support needed by particular groups.

### Conclusions

This technology-supported self-management telemonitoring and health counseling intervention did not improve the quality of life or clinical condition for patients with type 2 diabetes. There were significant differences between responders and nonresponders in the intervention group. Nonresponders reported being less familiar with the use of PCs, which suggests that those not accustomed to using computers had stopped participating in the study. More research is needed to target those not responding to telemedicine intervention and to understand how to design different telemedicine applications for different patient groups.

## Abbreviations

BMI: body mass index
GP: general practitioner
HbA1c: glycated hemoglobin
HDL: high-density lipoprotein
HRQoL: health-related quality of life
LDL: low-density lipoprotein
PC: personal computer
RH: renewing health
S-cholesterol: serum cholesterol
SD: standard deviation
SF-36: Short Form Health Survey

## Appendix

Multimedia Appendix 1 CONSORT-EHEALTH checklist.
